# An experimental design and implementation protocol for testing a dashboard for improving sustainable healthy food choice

**DOI:** 10.1016/j.mex.2025.103245

**Published:** 2025-02-22

**Authors:** Mariana Moncada de la Fuente, Ebenezer M. Kwofie, Prince Agyemang, Marie-Anne Dessureault, Ghina El Haffar, Laurette Dube, Stan Kubow, Valerie Orsat

**Affiliations:** a Bioresource Engineering Department, McGill University, Ste-Anne-de-Bellevue, H9X 3V9, Quebec, Canada; b Desautels Faculty Management, McGill University, 1001 Sherbrooke St W, Montreal, Quebec, Canada; c School of Human Nutrition, McGill University, Ste-Anne-de-Bellevue, H9X 3V9, Quebec, Canada

**Keywords:** Food choices, Environmental nutrition information, Dietary change, Sustainable diets, Testing of a mobile application in influencing consumer diet choices

## Abstract

Within the last decade, one of the crucial efforts to reduce environmental impact and improve consumers' health has focused on shifting food choices. In our previous study, the authors developed a customizable and adaptable Dashboard for Improving Sustainable Healthy (DISH) food choices. DISH leverages nudge and traffic-light labels to enable consumers to compare and envision the potential environmental, nutritional, and health impacts of their food choices before purchasing. An initial test among 112 individuals through an online survey revealed the potential of the tool to shift purchase intentions among consumers on a university campus. As part of a second phase in a series of consumer evaluations, we provide a step-by-step protocol followed to investigate the effectiveness of a version of DISH (McGill DISH) in stimulating subtle dietary changes on another university campus.

• Environmental nutrition information on DISH was communicated in simple but intuitive ways through multiple technological media (self-service kiosks and mobile applications) to stimulate dietary change.
• The study participants were randomly separated into treatment and control groups.
• We hypothesize that the participants in the treatment group are more likely to engage with food products that are more sustainable and healthier on the DISH application compared to the control group.

## Graphical abstract

**Figure:**
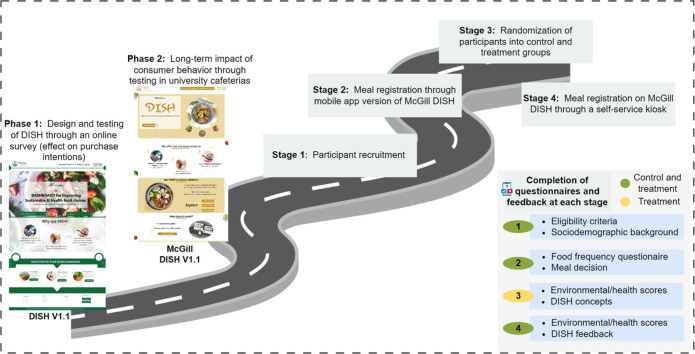

Specifications table

**Table:** 

Subject area:	Food Science
More specific subject area:	Sustainable healthy diets
Name of your method:	Testing of a mobile application in influencing consumer diet choices
Name and reference of original method:	Plamondon, G., Labonté, M.-È., Pomerleau, S., Vézina, S., Mikhaylin, S., Laberee, L., & Provencher, V. (2022). The influence of information about nutritional quality, environmental impact, and eco-efficiency of menu items on consumer perceptions and behaviors. Food Quality and Preference, 102, 104, 683.
Resource availability:	Not applicable

## Background

In our previous studies, the authors developed a decision support system, the Dashboard for Improving Sustainable Healthy (DISH) food choice, to stimulate consumers toward sustainable healthy choices [1]. DISH simultaneously maps out the health, nutrition, and environmental impact of meal choices from end users of the application. This enables consumers to envisage the potential impact of their choices before purchasing. Unlike existing tools, DISH employs behavioral change techniques coupled with traffic light labels and nudges to inform users about the nutritional and health performance of their selected meals. Users can see the environmental savings of meals chosen by examining the sustainability level of the available options. This feature was incorporated according to the protection motivation theory [2,3]. DISH's gamification module enables users to track the potential health and productive life gained/ lost effects from consuming a selected meal. In the early stage of the technological development of DISH, the environmental nutrition information of two fast-food menus, plant-based and animal-based burgers, was tested among 112 respondents from a university campus (University of Arkansas) through an online questionnaire survey. The results suggested that with an environmental nutrition score, less cognitive processing was required to make sustainable healthy choices. Among the 90.2 % of respondents with a predisposed purchase intention for animal-based burgers, 56.9 % reported a purchasing intent for plant-based burgers. More than 83 % attributed their decision to the environmental nutrition information provided on DISH. 64.3 % of respondents rated DISH as four stars or five stars, suggesting the perceived usefulness of the application. A statistical investigation of the results indicated that features of the DISH application, nudges, and awareness considerably influenced sustainable choices (sig < 0.001) [4].

As part of a second phase in a series of consumer tests and long-term impact evaluations, this article describes the experimental approach, tools, and implementation followed through by the authors to test the long-term impact of stimulating dietary change through the DISH application on McGill University campus. The core objective of the experimental design was to test whether “providing environmental nutrition and health information on foods using DISH installed on interactive platforms such as self-service kiosks and mobile phones can impact food choices”. Making a single change to an existing diet is more manageable for consumers than adopting an altogether new one, requiring less cognitive work to understand and remember and demanding less willpower if the change allows the consumption of a nutritionally equivalent meal. The article also highlights the anticipated risk and potential mitigation strategies and concludes on the potential benefits of preliminary results and insights gleaned from the test. The DISH version (McGill DISH) being tested is available online and can be accessed through any mobile device [5].

## Method details

### Methodological framework

**Fig. 1:**
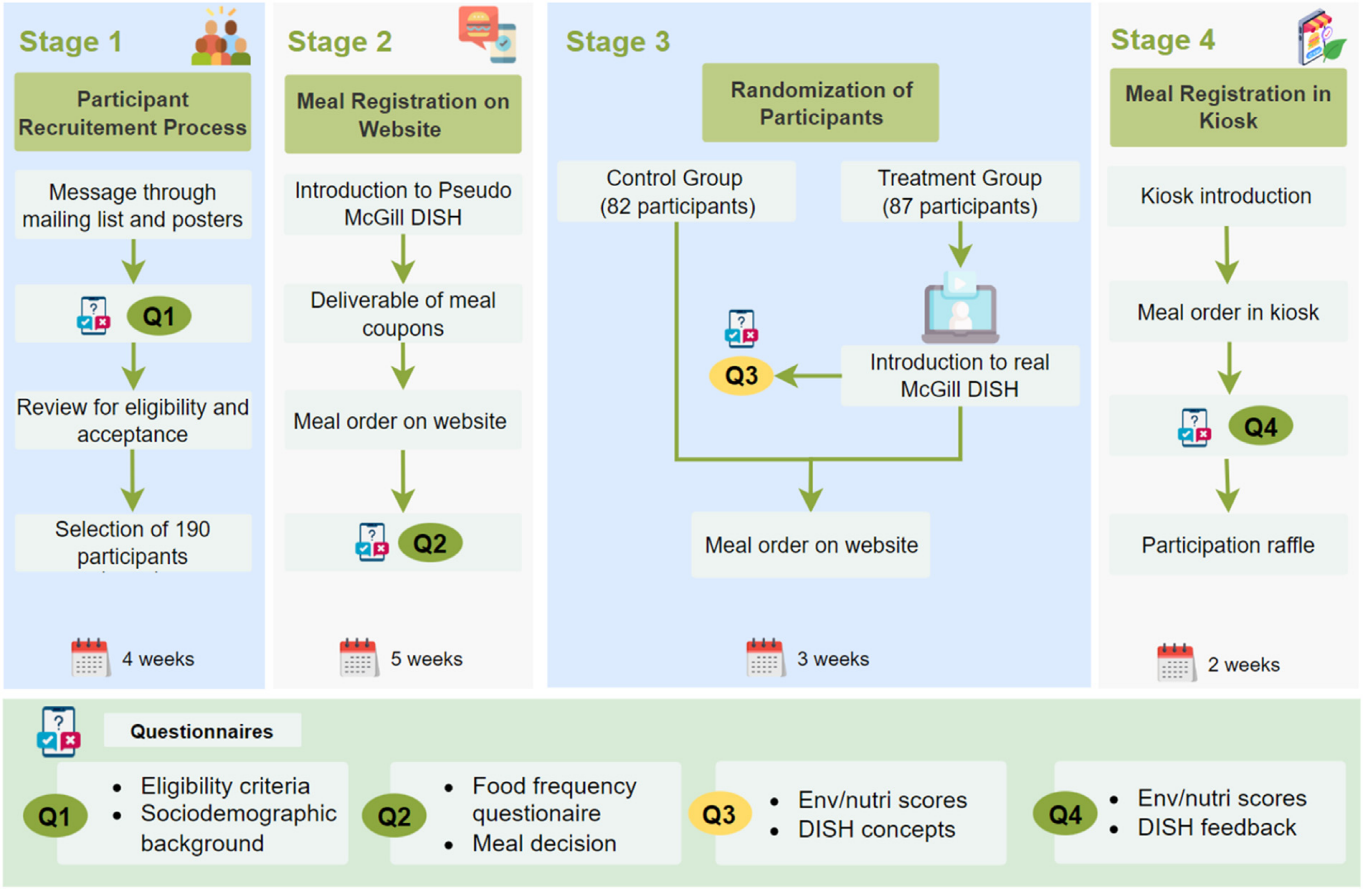
Conceptual framework of the experimental design and testing of DISH. The questionnaires in green (Q1, Q2, and Q4) are for the treatment and control groups. The questionnaires in yellow (Q3) are only for the treatment group.

The method framework was designed building on the work of Plamondon et al. [6] and [7]. The investigation to test the technology was divided into four stages (Fig. 1). The first stage involved determining the study environment, recruiting, and ensuring the acceptance of eligible participants. For this purpose, participants who met the eligibility criteria completed a sociodemographic background questionnaire.

In the second stage, participants accepted in the study received pseudo-user identifications (such as DISH-XA01), which were used to complete surveys and access the DISH application throughout the experimental period. Participants used their respective pseudo-user identifications to complete a food frequency questionnaire (FFQ) and a meal decision questionnaire. After submitting responses, participants received an infographic that explained how to access the pseudo version of McGill DISH (install the mobile version and log in using the respective pseudo identifications). Participants could access the DISH application via a website or mobile app at their preferred convenience. The pseudo version of the McGill DISH lacked environmental and nutritional indicators and was aimed at investigating the food patterns of participants before exposure. This investigation was conducted for five weeks, and participants received a weekly compensation of 7 Canadian Dollars (CAD) based on their purchase history.

In the third stage, participants were randomly separated into a treatment and control group. The control group continued using the pseudo-McGill DISH, and the treatment group used the authentic McGill DISH that displays all relevant features for stimulating dietary change. Each participant in the treatment group received information on the primary purpose of DISH and the different features included. Additional information was provided to participants in the treatment group on the metrics and scoring systems and the meaning of their environmental and nutritional scores (i.e., environmental and dietary scales of individuals and the average of selected meals). The participants in the treatment group reviewed their environmental and nutritional scores from previous meals and completed questionnaires regarding their user experience with the McGill DISH application. Two questionnaires were administered: the DISH concepts questionnaire and the Environmental/Nutritional questionnaire to understand the participants' decision-making when they registered their order, their reactions, and their thoughts about the impact of their dietary choices' information. At this stage of the experiment, we hypothesize that the participants in the treatment group are more likely to engage with food products that are more sustainable and healthier on the DISH application compared to the control group (**H_1_**). All participants continued using the mobile or web version of the application for three weeks. This enabled the project team to record the differences in the treatment group's decisions compared to the control group.

In the next stage of the experiment, both the control and treatment groups were exposed to the DISH application through a self-serving kiosk to place meal orders. The objective of the self-serving kiosk was to explore how reinforcement and communication of information on different platforms could yield a shift in their meal preferences and selections. This investigation was conducted over two weeks. Both control and treatment participants had access to different versions of the DISH (pseudo-DISH for the control group and authentic DISH for the treatment group). At this stage, we hypothesize that the exposure of the treatment group to the kiosk could effectively reinforce attitudes and intentions toward sustainable diet choices (**H_2_**).

At the end of the tenth week, all participants received new scores (environmental nutrition scores) and completed a questionnaire synonymous with the one administered in the fifth week. Aside from this, participants provided feedback and opinions about the DISH application on the self-service kiosks, the website, and the mobile app version. Overall, participants received compensation of 70 CAD for the study period. Participants who completed the entire experimental study (at least one meal purchase a week and completed all questionnaires administered) were entered into a draw to win a 100 CAD gift card. The sections below describe the activities conducted at each stage of testing the technology on the university campus.

### Study environment

The experimental design was implemented at the McGill University Macdonald campus in two cafeterias (Cafe Twigs and Ceilidh). The cafeteria provided all the ingredients and meals used in the study weekly. The study took place between August and December 2024.

### Criteria for participant recruitment

**Fig. 2:**
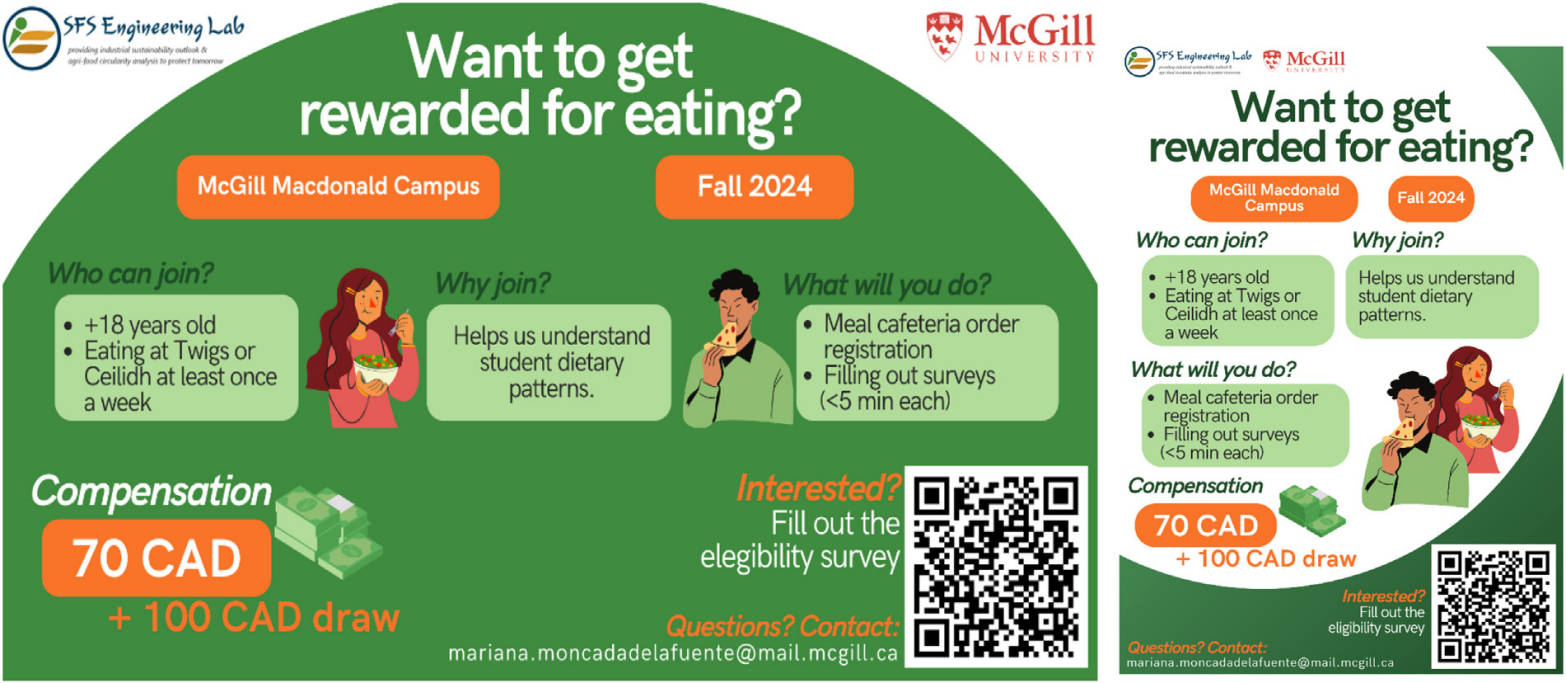
Design template for the poster used to recruit participants for the study.

The research team recruited 187 participants; however, by the fifth week, 169 participants remained. The remaining participants were randomly divided into 82 participants for the control and 87 for the treatment. Recruitment was made through the Macdonald Campus Student Society mailing list and posters on bulletin boards near participating cafeterias (Fig. 2). Respondents eligible to participate in the study were determined through a 2-minute online survey. The survey URL was embedded in a QR code that prospective respondents could scan directly. The survey briefly introduced the study, consent form, inclusion and exclusion criteria, and sociodemographic information. In this, respondents answered questions to identify whether they: (a) are students, staff, or both of the McGill University community, (b) dine at least once a week in at least one of the two participating cafeterias (Café Twigs and Ceilidh) on Macdonald Campus and had a favorable attitude towards 80 % of meals served, (c) are aged between 18 and 65 years, (d) had no food allergies or dietary restrictions that would prevent them from changing their dietary habits, (e) did not use any medications that could affect appetite sensation and food intake, and (f) most important must not have had any past or present form of eco-anxiety or eating disorder, which might affect their adherence to a strict dietary pattern. Lactating and pregnant women were excluded from the experiment as well.

### Study design and procedure

***Baseline characterization of participants:*** Upon completing the 2-minute survey, respondents received an email confirming their participation eligibility. Each participant completed a consent form and sociodemographic background before completing the food frequency questionnaire. The consent form provided the consequences of withdrawing from the study and other current and federal codes and regulations pertaining to human subject study. Each participant was provided with pseudo-identification to ensure their names were encoded during the qualitative research. However, the principal investigator had access to the pseudonyms so that in the event of a withdrawal or non-compliance with the activities requested for the study, participants could be removed from the study.

***Testing of McGill DISH through the web or mobile application:*** Participants' food choices in the cafeterias were monitored throughout the ten weeks. In the first five weeks, all participants used the pseudo-McGill DISH application for meal purchases. After this period, participants in the treatment group purchased food using the McGill DISH, while the control group continued to use the pseudo-McGill DISH application for meal ordering for five weeks. The application was accessible on smartphones, tablets, or any smart device. The participants in the treatment group were exposed to the environmental and nutritional indicators associated with each meal. Other relevant features, such as meal comparison and resource page, were visible for the treatment group (See the reference for the tested application [5]). At the same time, the contrary was done for the control group, who were exposed to the pseudo-McGill DISH. This provided an excellent opportunity to compare participants' dietary choices with and without introducing sustainability information, shedding light on any shifts in their meal preferences and selections. Furthermore, each participant purchased at least one meal a week at the participating cafeteria over a ten-week trial period. At the end of the tenth-week trial period, participants in the treatment group were requested to complete a DISH concept questionnaire to gauge their clarity regarding the terminologies used on the software and how they subsequently influence their choices during meal purchases.

***Testing of DISH through self-service kiosks:*** In the fourth stage, the DISH self-service kiosk was installed in the participating cafeterias. Here, participants in the control and treatment groups used self-service kiosks to purchase meals. This kiosk experiment was conducted over two weeks. Based on the participant pseudo-identification, each had access to a pseudo or authentic application version. This model provided an opportunity to explore the potential outcomes of sustainable healthy diets, particularly how the concept of reinforced health and environmental awareness through a different media in the treatment group will impact dietary choices as opposed to the control group with less exposure to the McGill DISH. The McGill DISH application installed on the kiosk was designed to allow guest participants access to the application. At the end of the experiment, participants from both groups completed a DISH concept and feedback questionnaire.

### Menu development and database

The cafeteria provided the research team with the ingredients and meals used in the study weekly or daily. Additionally, the Canadian import-export data and the provincial (Quebec) agricultural production and food consumption data were used to estimate the origin of the ingredients. All minerals and nutrient levels of the ingredients were obtained from the Canadian Nutrient File. The nutrients and minerals were normalized to energy functional units of 100 g and 100 kcal to ensure consistency with the Food Compass Scoring system, Health Nutritional Index score system, and midpoint environmental impact assessment results.

### Metrics and score

**Fig. 3:**
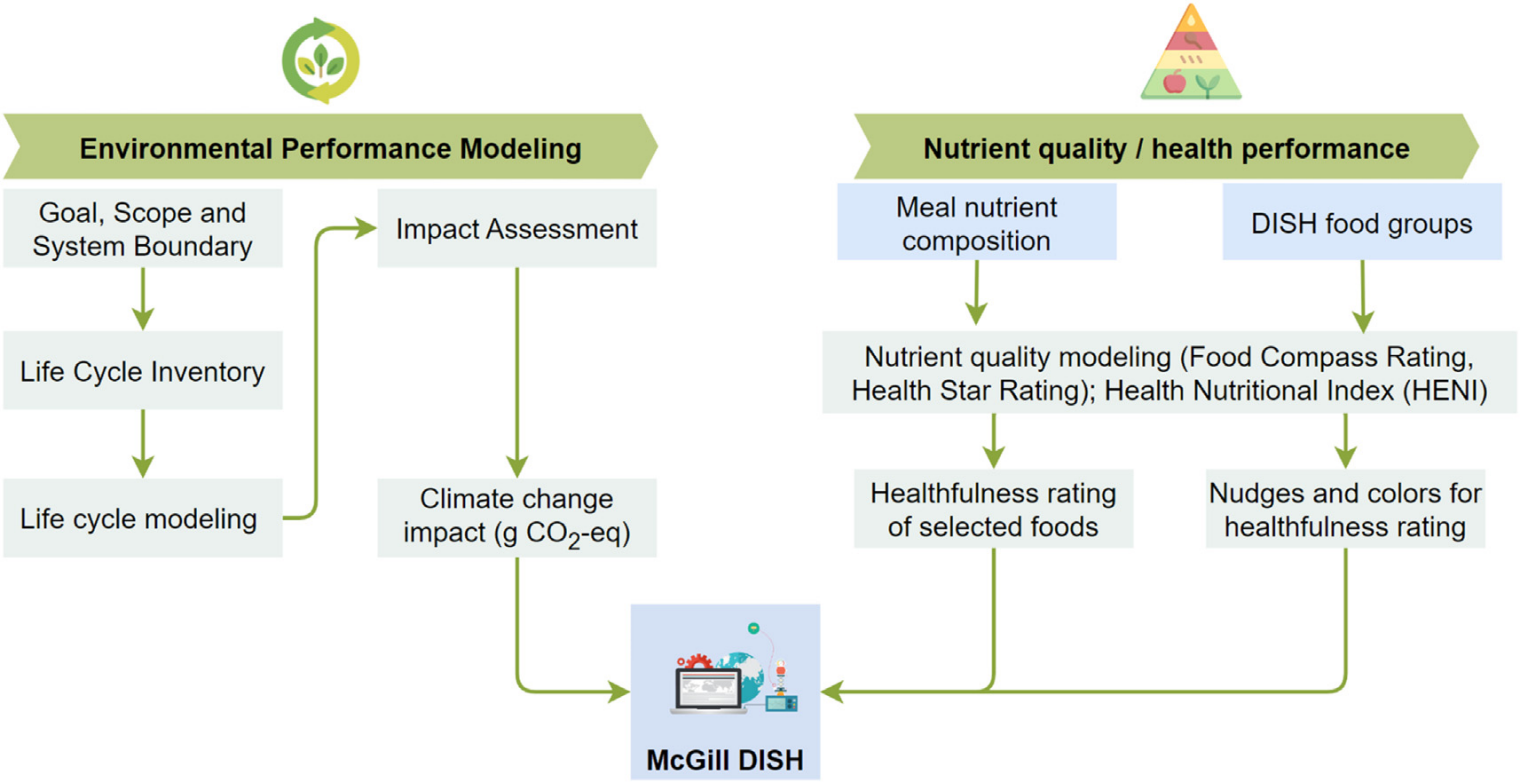
Metrics and score calculation for environmental performance modeling, nutrient quality and health performance, and meal database.

This section focuses on the calculations of the three metrics, which encompass nutritional profiling, health performance, and environmental impact assessment of meals available from the participating cafeterias. Fig. 3 summarizes the metrics and scores employed on the McGill DISH platform.

***Calculation Environmental Impact*:** The environmental impact assessment of the menu from the participating cafeterias was conducted using a life cycle approach following the ISO14040/44 method. The ISO 14040/44 outlines four primary steps: goal and scope definition, life cycle inventory, impact assessment, and interpretation of results. The environmental impact analysis of all menus was performed using an integrated Python-OpenLCA software version 2.0 interface [8] with Ecoinvent 3.7.1 [9] and Agribalyse [10]. For each menu, a function unit of 100 g was adopted. The ReCiPe 2016 Midpoint (H) v1.1 impact assessment methods and World 2010 (H/H) normalized method were used for the impact assessment. For the model developed for each meal, we assumed an average solid organic waste of 31.9 % for meal preparation according to the recommendations from Yu and Jaenicke [11].

***Nutrient Profiling:*** We build on the work of Mozaffarian et al. [12] to objectively profile the food items on menus of the participating cafeterias using the revised Food Compass Scoring (FCS) system. The FCS system employs 54 attributes across nine domains: nutrient ratios, vitamins, minerals, food ingredients, additives, processing, specific lipids, fiber, protein, and phytochemicals. It uniquely assigns a score of the healthfulness of the meals between 1 and 100 (with 100 being the most healthful and 1 being the least healthful). This could provide support for healthy eating behaviors and deliver personalized nutritional advice. A functional unit of 100 kcal was used to estimate the FCS for each meal. The FCS system was complemented by the Health Star Rating (HSR). The HSR was selected because it is one of the few that can easily be used to compare meals. Additionally, previous research and clinical trials by Todd et al. [13] and Detopoulou et al. [14] support the ease with which consumers can identify healthy and unhealthy foods with the HSR on the front-of-pack nutrition label. Thus, using the above nutrient profile methods supports the intuitive and easy communication of nutritional information to consumers.

The HSR of each meal on the menus was computed according to the Guide for Industry to HSR calculator [15]. First, baseline points were calculated for calories, saturated fat, total sugar, and sodium content per 100 g of the selected meal. The four components were considered due to their negative association with an increased risk of chronic diseases. Then, modification points for fruits, vegetables, nuts, legumes, protein, and fiber were calculated. Next, the health star score was estimated by computing the difference between the baseline and modifying points (see Eq. (1)). Finally, the score was converted to a star rating system based on a predefined scoring matrix and food category presented in the HSR calculator and guide [16]. The HSR algorithm generates a score rating from 0.5 (least encouraged) to 5.0 stars (most encouraged).

(1) HealthStarScore=baselinepoints−(Vpoints)−(Ppoints)−(Fpoints)…

where *V(points), P(points), and F (points)* are modifying points for vegetables, protein and fiber.

### Questionnaire design and administering

During the experiment trial, four questionnaires were administered from the recruitment stage to the focus group discussion stage, as shown in Fig. 1. All questions were administered through the McGill LimeSurvey, an online survey tool hosted on a McGill server and maintained by IT Services. The first set of questionnaires (eligibility criteria and sociodemographic background) was administered while soliciting participants for the study (Fig. 1). Feedback from ineligible respondents was discarded. In the eligibility criteria and sociodemographic survey, information such as age, gender, income, and education were collected. Next, a food frequency questionnaire was administered, which captured participants´ consumption of various foods and beverages. The objective was to determine participant's dietary patterns in and outside the selected cafeterias. After the food frequency questionnaire, a meal decision questionnaire capturing specific factors influencing behavior change was administered. This was based on the COM-B Model for Behavior Change developed by [17]. It provided an opportunity to understand the decision's background, such as the influence of money, social acceptance/trends/family, quality, health, comfort/convenience, and taste preference, along with to what extent they readily modify participants' eating habits. Two questionnaires were administered in the third stage of the experiment (Fig. 1). The questionnaires aimed to investigate the influence of environmental nutrition information on the treatment group and to analyze the effect and understanding of various concepts around the DISH application. All participants completed an Environmental/Nutrition scores questionnaire in week eight (Stage 3 of Fig. 1), respectively. Finally, at the end of week ten, a DISH feedback questionnaire was rolled out to participants to investigate user-friendliness, usability testing, and the DISH concept of both platforms. This survey measured the extent to which the end-users understood and were influenced by the terminologies employed. Besides feedback for potential enhancements, we investigated how reinforced information from the McGill DISH through a kiosk influenced food choice decisions for the treatment group, which had already used the website platform, as opposed to the control group, which had no exposure. The supplementary document presents the sets of questionnaires that were administered.

### Data collection and management

Data was collected via the McGill DISH kiosk and the web application. We integrated Google Analytics on the McGill DISH application to generate in-depth insights into user interactions across multiple mobile devices. We also monitored and analyzed user engagement metrics such as time spent on the platform, pages per session, user behavior, and the application's performance. Additionally, meal orders and purchases by participants were confirmed by cafeteria attendants using an administrative dashboard. All recorded meals from participants were stored in the Mongo DB database.

### Statistical analysis

**Fig. 4:**
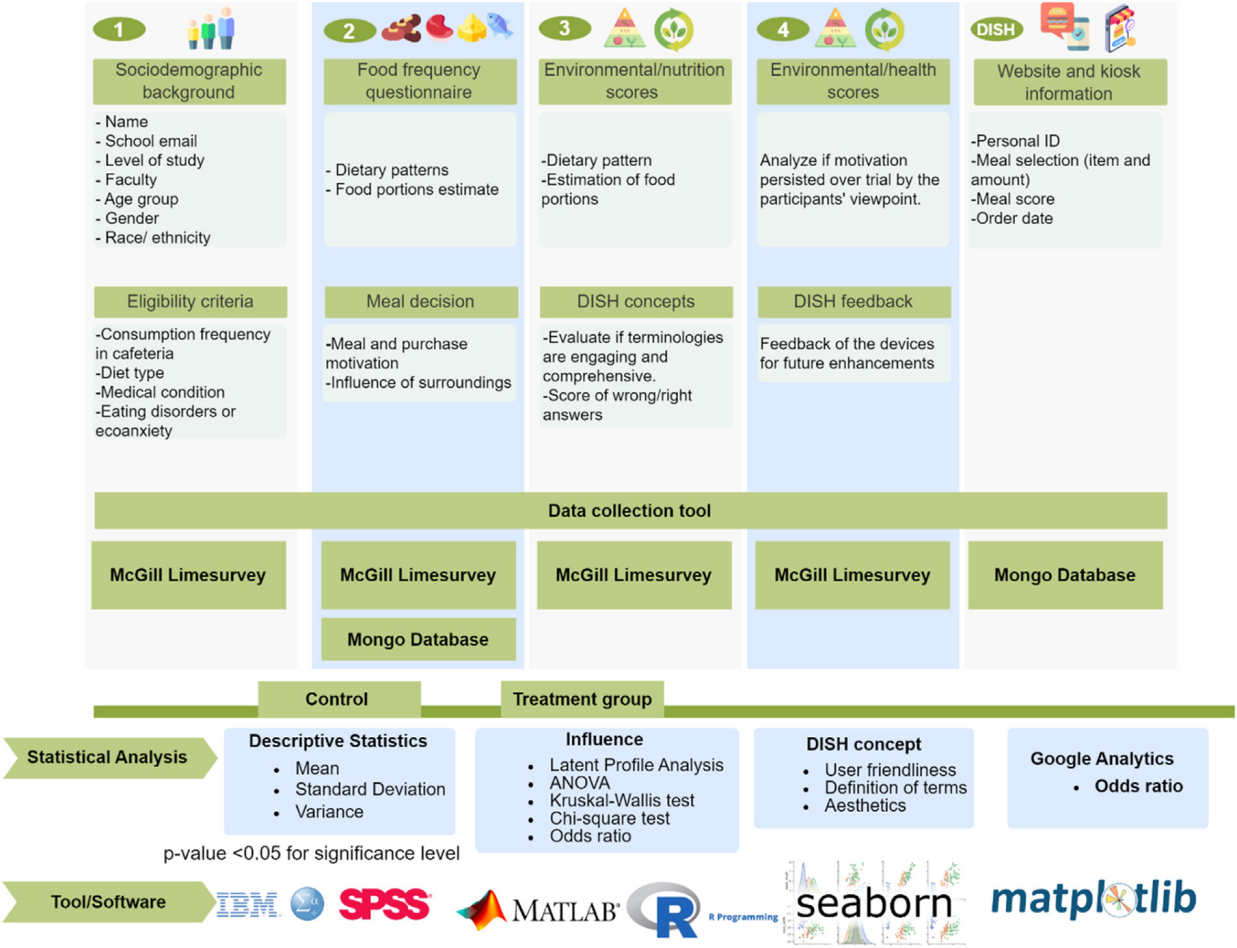
Characteristics of data, statistical analysis, and tools/software for the respective analysis.

Fig. 4 outlines the primary purpose of each questionnaire and the feedback provided, along with the methods, tools, and software used for statistical analysis. Descriptive statistical analysis will be calculated using the mean and standard deviation for the sociodemographic data and purchasing records from the trial sessions. A cross-tabulation analysis and analysis of variance will be used to examine the relationship and influence of demographic and purchase motivation variables on consumption frequency responses and meal purchases through the McGill DISH application. A baseline characteristic between the control and treatment groups will be compared using an analysis of variance, the Kruskal-Wallis test, and the Chi-square test, depending on the variable type. The significance level will be set at a p-value <0.05. Furthermore, we will employ latent profile analysis (LPA) to explore subgroups of participants who display similar patterns of multiple motivations for multiple consumers and to examine differences in subjective purchases across these subtypes [18,19].

The LPA works on the assumption that sample (residual) variance can be reduced by assuming a categorical latent variable that effectively subdivides the sample into ≥2 subgroups that are more homogeneous in terms of their patterns of variable means and (co)variance. Fortunately, ongoing development in the scientific field now allows LPA to be conducted in an open-source R-platform. Furthermore, odds ratios and a 95 % confidence interval will be calculated to measure the likelihood of choosing the meal with the most favorable score for the treatment group compared to the control group. These analyses will be done using MATLAB and Python statistical packages in combination with Microsoft Excel and the Statistical Package for Social Sciences software (SPSS by IBM Corp). The obtained analyzed data will be visualized using R-library ggplot2, Python-Seaborn and Matplotlib, and MATLAB data visualization packages.

## Method validation

### Meal database and characteristics

**Fig. 5:**
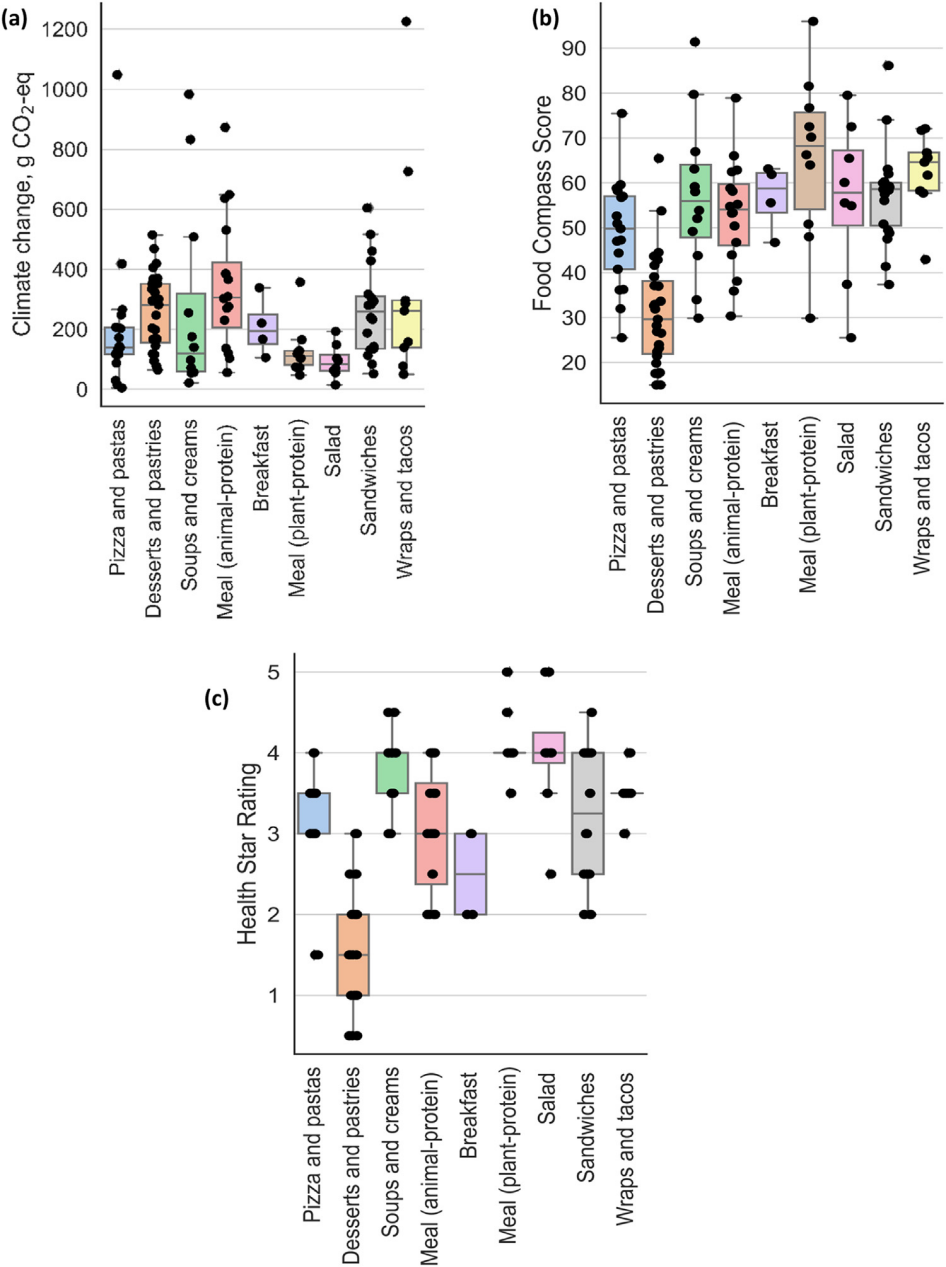
Trends in climate impact scores for meal purchases over the experiment period. (a) average climate score for all participant orders.

Fig. 5 presents the environmental nutrition characteristics of the meals provided by the two cafeterias over the experiment period. Overall, 122 meals were served over the 10 weeks of the experiment. Meals were categorized into nine groups: breakfast, wraps and tacos, pizza and pasta, sandwiches, meals with animal protein, meals with plant protein, salads, soups and creams, and desserts and pastries. Desserts and pastries were occasionally served, with 27 different lists of menu items served throughout the period of the experiment. On the contrary, meals in the category of salads (8 items) and breakfast (4 items) had the lowest list of items on the menu for the two participating cafeterias.

The climate change impact score for all meals served in the participating cafeterias ranged between 14.9 and 1225 g CO_2_-eq (Fig. 5(a)). Meals with animal-sourced protein and wraps and tacos resulted in relatively higher climate change scores compared to other meal categories. Meals with animal protein had scores ranging between 56.2 and 873 g CO_2_-eq (average score of 130 g CO_2_-eq), whereas wraps and tacos had scores ranging between 50.1 and 1226 g CO_2_-eq (average score 358 g CO_2_-eq). The climate change scores of wraps and tacos were relatively skewed by two meals, Chicken burrito wrap, and Pita steak, which were associated with scores of 726 and 1225 g CO_2_-eq. On the contrary, salad meals and meals with plant protein resulted in relatively low climate scores, with an average score of 93.2 and 130 g CO_2_-eq. The observed relatively lower impact score for meals with plant protein compared to animal protein corroborates with existing literature which indicates that plant-based diets are environmentally friendlier [20].

The HSR of meals served during the experiment period ranged between 0.5 and 5 stars for all meal categories (Fig. 5(c)). Meals with plant protein resulted in an average HSR score of 4.1, followed by salad meals with a score of 4.0 and soups and creams with a score of 3.8. The meal list in the category of dessert and pastries obtained an average score of 1.6, representing the lowest HSR score across all meal categories. Similarly, the FCS for all meals ranged between 25.5 and 96.0 (Fig. 5(b)). The list of menu items in meals with plant protein resulted in the highest average FCS of 65.6, whereas the list of menu items in the desserts and pastries yielded the lowest average FCS of 31.2. The results corroborate with existing literature. For example, [12] reported the FCS of food categories and food items well, with mean ± s.d. ranging from 17.1 ± 17.2 for savory snacks and sweet desserts to 81.6 ± 16.0 for legumes, nuts and seeds.

### Sociodemographic characteristics of participants

An estimated 396 people expressed an interest in participating in the experiment. However, 52.8 % of these potential participants were not recruited due to partial or incomplete filling of forms or not meeting the eligibility criteria. The remaining 47.2 % of people who expressed interest were recruited as participants at the beginning of the experiment. Table 1 presents the sociodemographic characteristics of participants who used the DISH application at the beginning of the experiment. Participants were required to have their meals at least once a week, though some did not strictly adhere to this requirement. The analysis revealed that the most predominant characteristics among participants were female (75.4 %), between 18 and 24 (73.3 %), white (39.5 %), undergraduate (63.1 %), and following an omnivorous diet (82.4 %). The “Others” category of race was mainly composed of mixed racial backgrounds. The staff and others in education include research assistants.

**Table 1:** Sociodemographic characteristics of participants involved in the current study.

Variable		Number	%
Sex	Male	40	21.4
	Female	141	75.4
	Non-binary	5	2.7
	Others/Prefer not to say	1	0.5
Age	Below 18	0	0
	18–24	137	73.3
	25–30	38	20.3
	31–40	12	6.4
	Above 40	0	0
Race	Asian	55	29.4
	Black/African American	21	11.2
	Hispanic	15	8
	White	74	39.5
	Others	22	11.8
Education	Undergraduate	118	63.1
	Graduate	62	33.2
	Staff/others	7	3.7
Dietary pattern	Vegetarian	3	1.6
	Omnivore	154	82.4
	Vegan	3	1.6
	Flexitarian	24	12.8
	Pescetarian	3	1.6

### Environmental-nutrition characteristics of meal purchase history

**Fig. 6:**
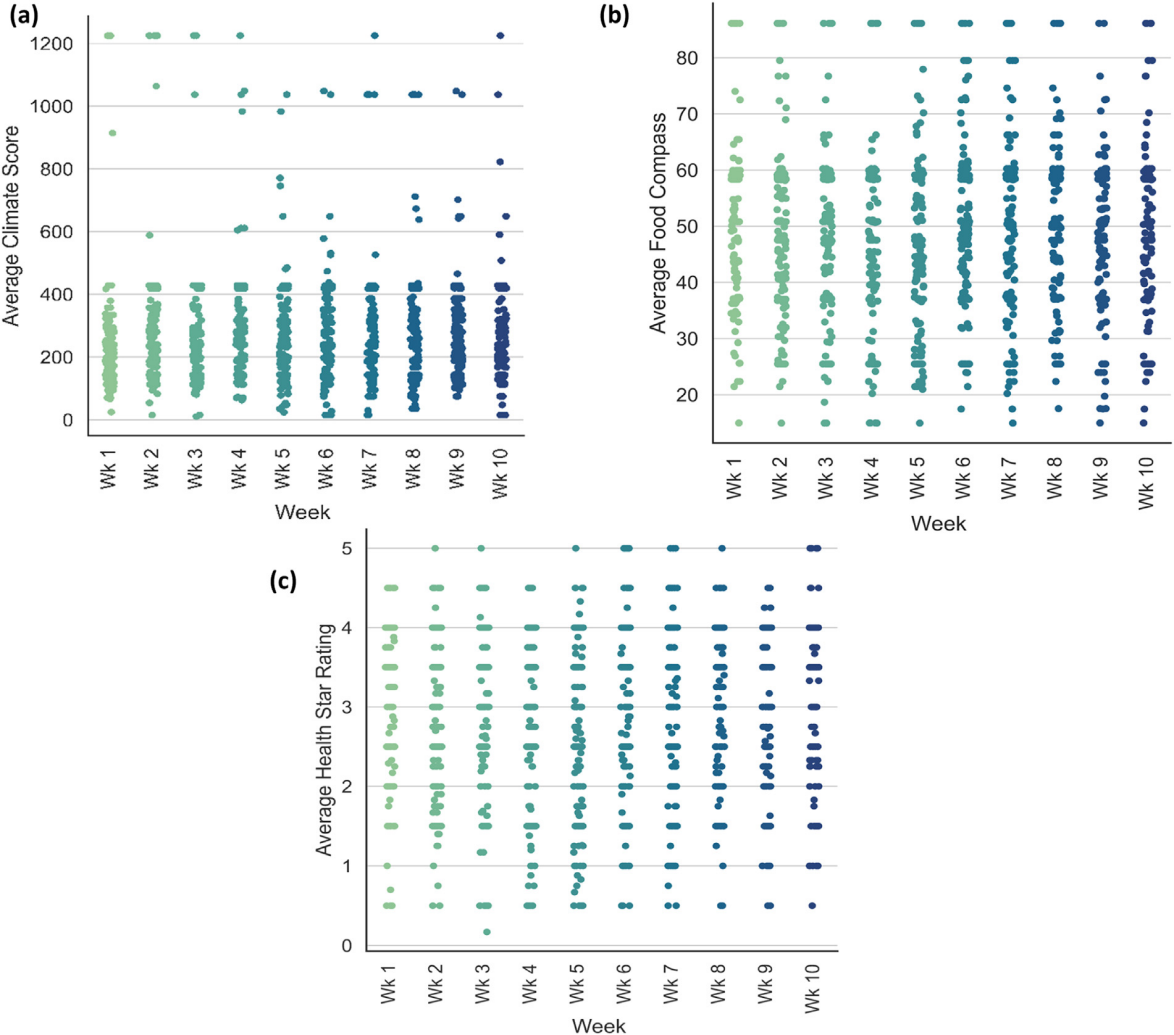
Trends in climate impact scores for meal purchases over the experiment period. (a) average climate score for all participant orders.

Fig. 6 presents the weekly trends in environmental nutrition metrics for meal orders placed by participants through the DISH application. Meal orders by participants were validated by cafeteria attendants using a customized cafeteria dashboard. The weekly trends prevent an average of all meal orders meal for each participant. It is important to highlight that the trends in climate change score, food compass score, and health star after week five are reported for both control and treatment groups. In Fig. 6(a), we observed that most meals ordered by participants had climate change scores between 20 and 600 g CO_2_-eq. Similarly, most meal orders were associated with an average food compass score between 20 and 80 and a health star rating between 1 and 4.5. Overall, we observed that participants

### Risk and mitigation strategies

Before the experiment, we anticipated that the implementation of this project could create a level of discomfort for individuals with a history of eating disorders, who might experience psychological distress due to food-related discussions. In addition, participants could experience emotional discomforts like feelings of guilt or environmental responsibility for eco-anxiety sufferers. Under the conditions where individuals experience such conditions, therapy groups and student wellness hubs on McGill Campuses will be engaged. However, the project team did not have such experience during the execution. Additionally, we anticipated participant withdrawal during the experiment. As a result, the compensation allocated to such participants was withdrawn accordingly. Also, during the self-service kiosk implementation in the participating cafeterias, we anticipate non-participants will use it to make meal orders. Hence, a guest entry button was created, granting individuals access to the McGill DISH to place meal orders without necessarily being part of the experiment.

## Conclusion

The study sets out to report the design of an experiment and an implementation plan for testing a novel decision support system known as the McGill DISH. The data generated from the experimental design implementation plan will inform whether communicating environmental, nutritional, and health indices associated with meals through different media in an institutional setting could stimulate individuals to make more sustainable diet choices. The study will generate insights into how point-of-purchase information provided to consumers and accessible through smartphone devices and self-serving kiosks could support more sustainable food choices. Additionally, insights into behavioral and purchase motivation drivers and their role in consumer food choices were generated, which could inform future studies and the testing of DISH in different formats and institutions. Additionally, the implementation framework could be adapted to future studies as the authors consider the testing of an adaptable DISH technology in different institutional and public settings. We believe pushing for a universally accepted diet for people is unrealistic and unethical. However, subtle diet changes through technologies such as DISH offer an opportunity to mitigate climate change, improve human health, and promote sustainable, healthy food choices.

## Limitations

Not applicable

## Ethics statements

The study received ethical approval from the Research Ethics Board Office (REB #23-09-072). Each of the study team members was certified by the Tri-Council Policy Statement: Ethical Conduct for Research Involving Humans (TCPS 2). Participants consented before completing all surveys administered during the experiment.

## CRediT authorship contribution statement

**Mariana Moncada de la Fuente:** Conceptualization, Methodology, Writing – original draft, Writing – review & editing. **Ebenezer M. Kwofie:** Funding acquisition, Supervision. **Prince Agyemang:** Conceptualization, Methodology, Writing – original draft, Writing – review & editing. **Marie-Anne Dessureault:** Conceptualization, Methodology. **Ghina El Haffar:** Conceptualization, Methodology. **Laurette Dube:** Supervision, Conceptualization. **Stan Kubow:** Supervision, Conceptualization. **Valerie Orsat:** Supervision, Writing – original draft, Writing – review & editing.

## Declaration of competing interest

The authors declare that they have no known competing financial interests or personal relationships that could have appeared to influence the work reported in this paper.

## Data Availability

Data used in this research will be provided upon request..

## Appendix Supplementary materials

Image, application 1

Image, application 2
