# Supplementary material for: An experimental design and implementation protocol for testing a dashboard for improving sustainable healthy food choice
Source: MethodsX. 2025 Feb 22;14:103245. doi: 10.1016/j.mex.2025.103245 (PMC11919336; doi:10.1016/j.mex.2025.103245)
Supplement: Supplementary file 1 [file mmc1.docx]

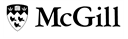


**Participant Consent form**

**Researchers:** Mariana Moncada de la Fuente, MSc. Bioresource Engineering student, [mariana.moncadadelafuente@mail.mcgill.ca](mailto:mariana.moncadadelafuente@mail.mcgill.ca)

**Supervisor:** Dr. Ebenezer Miezah Kwofie. Assistant Professor in Bioresource Engineering. T: 514-398-7776, [ebenezer.kwofie@mcgill.ca](mailto:ebenezer.kwofie@mcgill.ca)

**Title of Study:** A Pilot Implementation of an Online Simulator for Meal Decisions (REB #23-09-072)

**Sponsors:** The research fund is sponsored by the Danone Institute North America.

**Purpose of the study:** The study intends to comprehend the current frequency of meal orders in the McGill cafeterias and the usual meal choices of the McGill community. In addition, we aim to comprehend the impact of providing different platforms to facilitate meal orders and compare which one has a better response for consumers.

**Study procedures:** As a participant, you must consider that the experiment will be held for ten weeks in Fall 2024, between September and November. During this period, you will be requested to register on the website and self-service kiosk for every meal you consume in the cafeterias of the Macdonald campus, Twigs, and Ceilidh. In addition, 4 different questionnaires and feedback will be given, each one lasting around 5-10 minutes. To consent to your participation in this study, it is crucial that you sign below, stating that you have read the information provided and agree to participate in the study.

**Voluntary participation:**

Your participation in this study is entirely voluntary. You may decline your participation, answer any question, take part in any procedure, or choose to withdraw at any time, and no explanation will be requested. In case of withdrawal, you must contact the main researcher directly via email ([mariana.moncadadelafuente@mail.mcgill.ca](mailto:mariana.moncadadelafuente@mail.mcgill.ca)).

**Potential Risks:**

There are no anticipated risks to you by participating in this research.

**Potential Benefits:**

Participating in the study will not directly benefit you. However, the study aims to comprehend meal choices within the McGill community. By doing so, you can gain insights into dietary patterns and contribute to a better understanding of factors influencing food choices. The anticipated value of this study lies in its potential to comprehend whether presenting outcomes engagingly and interactively can shift consumers' meal selections.

The results of this study will be disseminated through various channels to maximize their impact. They will be made available on the dashboard website and social media handles, ensuring accessibility to a broad audience. The findings will be shared through academic publications and presentations, reaching the academic community and potentially influencing future research and policy discussions.

**Compensation:**

Compensation will be provided weekly only after you complete the required tasks, which include meal registration and, when applicable, questionnaires. You will receive 7 CAD per week for 10 weeks, totaling 70 CAD. You are highly encouraged to register more than one meal per week. Registering additional meals will not increase your monetary compensation, but it will give you an entry into a draw to win 100 CAD at the end of the study. If you decide to withdraw from the study before its completion, you will keep the compensation you have already received, but you will not be eligible for any further scheduled payments, nor will you be included in the final draw, which is reserved for participants who complete the study. To receive monetary compensation, an e-transfer will be sent to your institutional email address by the project coordinator.

**Confidentiality:**

All data collected will be confidential and will not be linked to individuals. Your full name, email address, and username are required only for contacting you and providing compensation and will not be included in any data collection instruments. Future publications will contain no participant-related information. Your age group, race or ethnicity, affiliation, and gender will be used to identify patterns in the statistical results but will not be directly linked to your identity. The data analysis aims to understand the sociodemographic influence on meal choices while maintaining participant confidentiality. To protect participants' identities during recruitment and data collection, you will be allowed to create a personal username that can be unrelated to your personal identity.

The raw data will be accessible only to the supervisor, the project coordinator, and two additional students, all members of the research team. The supervisor will be responsible for long-term data storage. All data will be shared as analyzed data, not individual datasets. You have the right to consult any of your personal information gathered for the purposes of this study and to have it corrected, if necessary, by contacting a member of the research team ([mariana.moncadadelafuente@mail.mcgill.ca](mailto:mariana.moncadadelafuente@mail.mcgill.ca)). All project information will be securely stored for seven years in the research supervisor's McGill One Drive (cloud storage). This data, including non-identified data, may leave the province as the McGill One Drive is a backup server outside of Quebec.

The code key, which links your name with your username, will be destroyed one year after the experiment concludes. Consequently, data withdrawal will not be possible after this period. If you choose to withdraw during the experiment or before the one-year timeframe, you can decide whether your provided information can be retained for analysis or discarded.

**Dissemination of Results:**

You will provide consent that no personal information will be used during the experiment and that your questionnaire answers will be used for analysis in the experimental study without any relation to your personal identity. The information received at the kiosk for the population-based study will include a box indicating that the meal order will be recorded for the study.

The information provided will be the overall food choices on campus, excluding any personal information. This analyzed data will be shared with relevant authorities on campus, such as the McGill Sustainability Office and McGill Hospitality, to identify solutions for a more sustainable institution. Additionally, the results are expected to be published in various media, such as academic publications, to share the impact of the study. No raw data will be shared with other researchers or users.

**Questions:**

For any additional questions, feel free to contact the project coordinator, Mariana Moncada de la Fuente, via email ([mariana.moncadadelafuente@mail.mcgill.ca](mailto:mariana.moncadadelafuente@mail.mcgill.ca)) or the research supervisor ([ebenezer.kwofie@mcgill.ca](mailto:ebenezer.kwofie@mcgill.ca)). If you have any ethical concerns or complaints about your participation in this study, and want to speak with someone not on the research team, please contact the Research Ethics Board Office, Daniel.tesolin@mcgill.ca or 514-398-5410, citing REB file number 23-09-072

**Consent:**

After reviewing the above information and providing your consent to participate, please check the following box. You are strongly advised to read this content thoroughly before confirming your consent. Upon completion of this consent form, a copy of your responses will be sent to your email and the research team. We recommend downloading or saving this information for your records. Your agreement to participate in this study does not relinquish any of your rights or exempt the researchers from their responsibilities. As a security measure, a member of the Research Ethics Board will have access to this information. Giving consent implies the condition that compensation, in the event of withdrawal, will be provided based on the stage at which you choose to exit. In addition, you consent to your anonymized information to be retained for potential future use in research.

You consent to participate in this research study and perform the associated tasks.

You consent to receiving e-transfers for monetary compensation using your institutional email. (Due to security measures, the institutional email is the only one we can use.)
