# Supplementary material for: An experimental design and implementation protocol for testing a dashboard for improving sustainable healthy food choice
Source: MethodsX. 2025 Feb 22;14:103245. doi: 10.1016/j.mex.2025.103245 (PMC11919336; doi:10.1016/j.mex.2025.103245)
Supplement: Supplementary file 2 [file mmc2.docx]

**An Experimental Design and Implementation Protocol for Testing a Dashboard for Improving Sustainable Healthy Food Choice**

*Mariana Moncada de la Fuente^1^, Ebenezer M. Kwofie^,1^, Prince Agyemang^1^, Marie-Anne Dessureault^1^, Ghina El Haffar^2^, Laurette Dube^2^, Stan Kubow^3^, and Valerie Orsat^1^*

*^1^Bioresource Engineering Department, McGill University, Ste-Anne-de-Bellevue, H9X 3V9, Quebec, Canada*

*^2^Desautels Faculty Management, McGill University, 1001 Sherbrooke St W, Montreal, Quebec, Canada*

*^3^School of Human Nutrition, McGill University, Ste-Anne-de-Bellevue, H9X 3V9, Quebec, Canada*

**Correspondence: prince.agyemang@mail.mcgill.ca*

**Questionnaire Series 1**

- 1. **Eligibility criteria and sociodemographic background**

**McGill Limesurvey**

The study's objective is to understand meal frequency consumption in campus cafeterias. To participate, consider the following criteria: participants must be McGill undergraduate and graduate students who eat at least once a week in Twigs and/or Ceilidh at the Macdonald campus. No specific sociodemographic background is required. The students should not have any dietary restrictions or food allergies. Students should not be on medication or any conditions that might prejudice them by changing dietary meals. Participants cannot have a record of any eating disorder or feel targeted by any food. Participants with eco-anxiety are also excluded. Participants must agree to the consent form given prior to the experiment.

If you meet the eligibility criteria and are interested in participating, please provide the following information:

1. Name
2. Student email address
3. Which of the following do you relate to?
4. Undergraduate (b) Graduate (c) Professor (d) Other:_____
5. Which faculty are you affiliated with?

_____________

1. In which age group are you in?
2. Below 18 (b) 18-24, (c) 25-30, (d) 31-40, (e ) above 40.
3. Please, specify your gender:
4. Male, (b) female, (c) non-binary/third gender (d) Prefer not to say
5. Please specify your race or ethnicity:
6. White (b) Black/African American (c) Asian (d) Hispanic (e) Other:__ (f ) Prefer not to say
7. On average, how much money do you typically spend per month on food? Please provide an approximate amount. If you are unsure, please provide your best estimate.
8. Less than $100 (b) $100 - $300 (c) $301 - $500 (d) More than $500 (e) Prefer not to answer
9. Do you or would you be willing to eat at least once a week at Twigs and/or Ceilidh at the Macdonald campus?
   1. Yes b. No
10. How would you describe your diet?
11. Omnivore (b)Pescetarian (c) Flexitarian (d) Vegetarian (e) Vegan
12. Do you have any dietary restrictions or food allergies?
13. Yes (specify):_____ (b) No
14. Do you have any medical condition or take any medication that do not allow you to change your diet?
15. Yes (b) No
16. Do you have any eating disorder or take any medications that can affect appetite sensation and food intake?
17. Yes (b) No
18. Do you have any eco-anxiety (a chronic fear of environmental doom) related issues?
19. Yes (b) No
20. Would you feel comfortable monitoring your meal orders in the selected cafeterias over a 3 to 4-month lapse?
21. Yes (b) No

**Questionnaire Series 2**

## 2.1 Food Frequency Questionnaire

1. How often do you eat meat and cured meats?
   1. Every day b. Every two days c. Once or twice a week d. Once or twice a month e. Never
2. How often do you eat red meat?
   1. Every day b. Every two days c. Once or twice a week d. Once or twice a month e. Never
3. How often do you eat fish and shellfish?
   1. Every day b. Every two days c. Once or twice a week d. Once or twice a month e. Never
4. How often do you eat eggs?
   1. Every day b. Every two days c. Once or twice a week d. Once or twice a month e. Never
5. How often do you eat pulses (lentils, chickpeas, beans, peas…)?
   1. Every day b. Every two days c. Once or twice a week d. Once or twice a month e. Never
6. How often do you eat nuts?
   1. Every day b. Every two days c. Once or twice a week d. Once or twice a month e. Never
7. How often do you eat other plant proteins (tofu, tempeh…)
   1. Every day b. Every two days c. Once or twice a week d. Once or twice a month e. Never
8. How processed are the proteins you usually eat?
   1. No processing whatsoever b. Low level of processing (tofu, canned beans...) c. High level of processing (industrial sausages...)
9. How often do you eat non-processed cereals?
   1. Every day b. Every two days c. Once or twice a week d. Once or twice a month e. Never
10. How often do you eat processed cereals?
    1. Every day b. Every two days c. Once or twice a week d. Once or twice a month e. Never
11. How often do you eat dairy products?
    1. Every day b. Every two days c. Once or twice a week d. Once or twice a month e. Never
12. How many servings of non-processed (fresh) fruits and vegetables do you eat every day?
    1. 0 servings b. 1-2 servings c. 3-4 servings d. 5+ servings
13. How many servings of processed fruits and vegetables do you eat every day?
    1. 0 servings b. 1-2 servings c. 3-4 servings d. 5+ servings
14. How often do you eat sweets (chocolate, cookies, cakes, pastries...)?
    1. Everyday b. Every two days c. Once or twice a week d. Once or twice a month e. Never
15. Rank the following drinks on a scale of 1-5 (1 being I never drink it and 5 being I drink it every day).
    1. Water b. Packaged fruit juices c. Fresh fruit juices and smoothies d. Sweetened beverages (soda, sweetened tea) e. Alcoholic beverages f. Coffee

## Meal decision (COM-B method)

1. Do you follow any diet or nutritional regimen?
   1. Yes (drop-down to vegetarian, pescetarian, keto, flexitarian, Mediterranean, intermittent fasting, other) b. No
2. To what extent do you monitor the food you eat?
   1. Not at all b. Sometimes c. Most of the times d. All the time
3. On a scale of 1-5, do you consciously try to eat healthy foods? (1 being not at all and 5 being all the time)
4. On a scale of 1-5, do you consciously try to eat environmentally friendly foods? (1 being not at all and 5 being all the time)
5. How often do you consume in the Macdonald Campus’ cafeterias (Twigs and Ceilidh)?
   1. Every day b. 3-4 times a week c. 1-2 times a week d. Less than once a week
6. How often do you eat outside of your home (restaurants, school cafeteria, or at other people’s places)?
   1. Every day b. 3-4 times a week c. 1-2 times a week d. Less than once a week
7. To what extent do the people who live with you (roommates, partners, family) help and encourage you to eat in a healthy way? (1 being not at all and 5 being all the time)
8. To what extent do the people who live with you (roommates, partners, family) help and encourage you to eat in an environmentally friendly way? (1 being not at all and 5 being all the time)
9. How much do your friends and family who don't live with you encourage you to eat healthy? (1 being not at all and 5 being all the time)
10. How much do your friends and family who don't live with you support you in eating environmentally friendly? (1 being not at all and 5 being all the time)
11. On a scale of 1-5, how expensive do you think healthy food is compared to unhealthy food? (1 being cheaper and 5 being much more expensive)
12. On a scale of 1-5, how expensive do you think environmentally conscious food is compared to environmentally damaging food? (1 being cheaper and 5 being much more expensive)
13. What are the barriers for you to eat healthy food? Select all that apply.
    1. Budget b. Time c. Lack of knowledge d. Lack of care e. Other: please precise
14. What are the barriers for you to eat environmentally friendly food? Select all that apply.
    1. Budget b. Time c. Lack of knowledge d. Lack of care/motivation e. Other: please precise
15. To what extent do you think the food you eat impacts your health? (1 being no impact whatsoever and 5 being very impactful)
16. To what extent do you think the food you eat impacts the environment? (1 being no impact whatsoever and 5 being very impactful)
17. To what extent do you want to eat healthier food? (1 being I do not want to eat healthier food and 5 being I really want to eat healthier food)
18. To what extent do you want to eat more environmentally conscious food? (1 being I do not want to eat more environmentally conscious food and 5 being I really want to eat more environmentally conscious food)

# Questionnaire Series 3

## Environmental and Nutrition Scores (before)

1. Were you previously aware of the environmental and nutritional impact of your food and diet choices? Yes/ No
2. Do you believe the scores you received fall within a favorable range for your health and the environment? Environment: Yes/ No Health and Nutrition: Yes/ No
3. How does your score make you feel? Choose one:

(a)Indifferent (b) Overwhelmed (c) Hopeful (d) Triggered (e) Other (specify):

1. Which piece of information engaged you the most?

(a) Life minutes gained/lost (b) cost to the environment (c) comparison with other recruiters (d) All of the above

1. Do you believe using the McGill DISH platform will influence your future meal selections? Choose one:
2. Definitely not (b) Probably not (c) Might or might not (d) Probably yes (e ) Definitely yes

## DISH Concepts

## (Correct answers are highlighted in red)

1. What is the McGill DISH?
2. A toolbox used to determine the amount of sugar the McGill community is consuming (b) a toolbox that helps cafeterias record meal purchases to understand what the McGill community likes the most **(c) an intuitive and transparent toolbox that aims to inform consumers about the outcomes of their food selection before and after purchasing, encouraging users to make informed nutritional and ecological decisions.**
3. What are the benefits of using McGill DISH?
4. Provide information about meals in a more interactive way (b) guide users to choose a meal based on their consumption goals (c) compare the environmental and health scores within the available option in the McGill cafeterias **(d) All of the above**


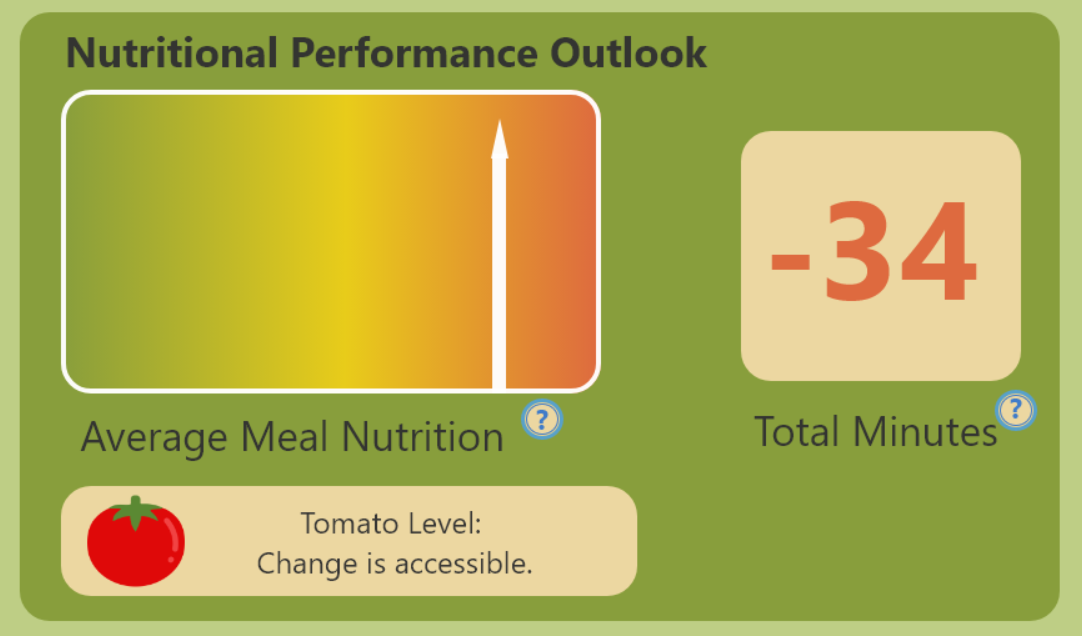


1. What do total minutes refer to?
2. Minutes used to prepare the product (b) **productive minutes of life gained or lost** (c) minutes of meal digestion.
3. What does the average meal nutrition refer to?
4. **How healthy the product is** (b) how likable it is for the team members (c) the level of protein the product has.
5. Which would be a healthier option?
6. Less total minutes (b) **More total minutes**

**
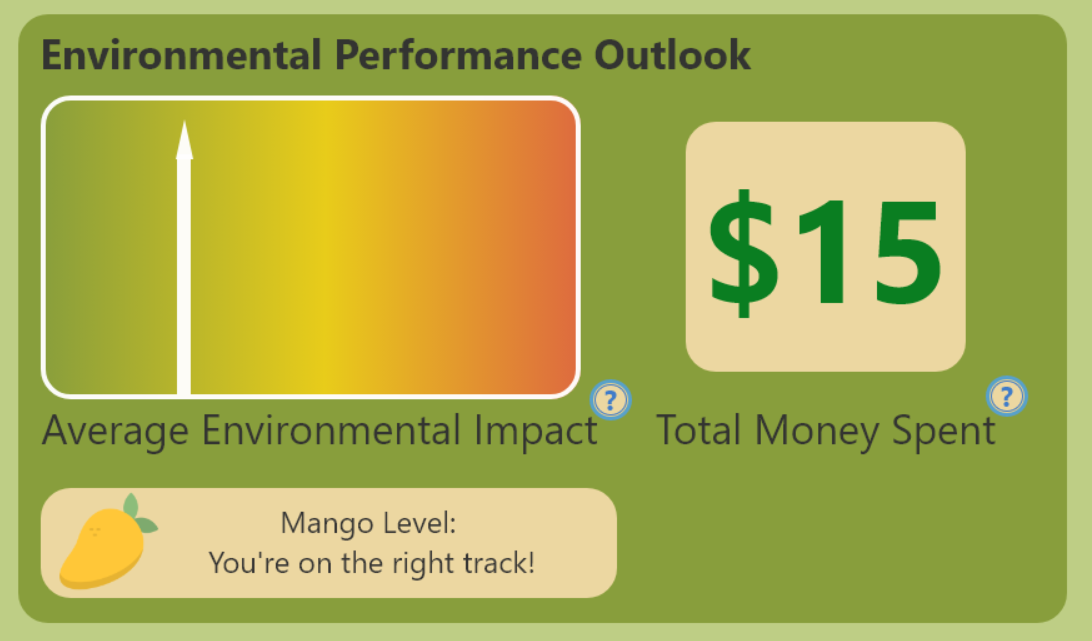
**

1. What does total money spent refer to?
2. What it cost the cafeteria to make 10 servings **(b) cost to remediate the environment after producing the food** (c) price of the ingredients
3. What does average environmental impact refer to?
4. Depends on how many plant-based products the meal has **(b) The level of how environmentally friendly the product is** (c) How many steps were necessary to obtain the product.
5. Which would be more environmentally friendly?
6. **Cheaper cost** (b) more expensive cost
7. If you would like to improve any of these scores, what can you do?
8. **Change meal** (b) Ask people from the cafeteria (c) Nothing
9. Is there any concept that was not understandable?
10. Yes______ (b) No

# Questionnaire Series 4

# Environmental and Nutrition Scores (after)

1. Do you believe the scores you received fall within a favorable range for your health and the environment? Environment: Yes/ No Health and Nutrition: Yes/ No
2. How does your score make you feel? Choose one:

(a)Indifferent (b) Overwhelmed (c) Hopeful (d) Triggered (e) Other (specify):

1. Which piece of information engaged you the most?

(a) Life minutes gained/lost (b) cost to the environment (c) comparison with other recruiters (d) All of the above

1. Do you believe using the McGill DISH platform will influence your future meal selections? Choose one:
2. Definitely not (b) Probably not (c) Might or might not (d) Probably yes (e ) Definitely yes

## DISH Feedback

### Self-Service Kiosk

1. Do you trust the information presented on the simulator?
   1. Definitely not (b) Probably not (c) Might or might not (d) Probably yes (e ) Definitely yes
2. Would you recommend that more information should be added to the simulator?

(a) Yes: ______ (b) Maybe (c) No

1. Have you used any simulator like this?
   1. Definitely not (b) Probably not (c) Might or might not (d) Probably yes (e ) Definitely yes

Which areas do you think the application can improve for a better user experience?

_________________

1. Overall, how will you rate this simulator? (1 being the worst and 5 the best)
2. 1 (b) 2 (c) 3 (d) 4 (e) 5
3. Rate the following criteria of the DISH platform on a scale from 1 to 5 (1 being totally disagree and 5 totally agree):

- The platform made my meal decision to be easier, and the things are done effectively
- The platform kept me interested and involved, making it an engaging experience.
- The platform is easy to use, and the information provided is understandable, allowing me to choose my meals effortlessly.
- The visual aids are appealing, and I like the aesthetics.
- The platform presents information in a way that is easy to understand.
- The information provided was interesting, and I remember it when I want to purchase a meal, even if I do not use the platform.

1. Please provide any suggestions on the self-service kiosk:

__________________

### Website

1. Do you trust the information presented on the website?
   1. Definitely not (b) Probably not (c) Might or might not (d) Probably yes (e ) Definitely yes
2. Would you recommend translating this website into a mobile application that would be more useful to guide your sustainable decisions in the future?
3. Yes (b) Maybe (c) No
4. Would you recommend that more information should be added to the simulator?

(a) Yes: ______ (b) Maybe (c) No

1. Have you used any simulator like this?
   1. Definitely not (b) Probably not (c) Might or might not (d) Probably yes (e ) Definitely yes
2. Which areas do you think the application can improve for a better user experience?

______________

1. Overall, how will you rate this platform? 5 (1 being the worst and 5 the best)
2. 1 (b) 2 (c) 3 (d) 4 (e) 5
3. Rate the following criteria of the DISH platform on a scale from 1 to 5 (1 being totally disagree and 5 totally agree):

- The platform made my meal decision to be easier, and the things are done effectively
- The platform kept me interested and involved, making it an engaging experience.
- The platform is easy to use, and the information provided is understandable, allowing me to choose my meals effortlessly.
- The visual aids are appealing, and I like the aesthetics.
- The platform presents information in a way that is easy to understand.
- The information provided was interesting, and I remember it when I want to purchase a meal, even if I do not use the platform.

1. Please provide any suggestions on the website:

# Administering of Questionnaires

Throughout the experiment, four series of questionnaires and surveys will be provided:

1. **Recruitment**
   1. **Eligibility criteria and Sociodemographic background:** register the demographic background of each participant and understand if the results vary depending on their background.
2. **Food Daily Choices**
   1. **Food Frequency Questionnaire:** observe the difference in participants' daily consumption patterns from the cafeteria meals.
   2. **Meal Decision (COM-B model)**: explores the motives behind meal purchases, considering possible scenarios such as taste preference, price, convenience, trends, health, and environmental impact.
3. **Engagement and influence of DISH**
   1. **Environmental and Nutrition Scores (before):** provide information about the environmental and health impact of dietary decisions and analyze if a personal score influences their meal choices. Additionally, participants will be asked about their typical consumption of animal and plant-based foods and their knowledge of these choices' health and environmental impacts.
   2. **DISH Concepts:** evaluate if the information and terminologies provided are understandable and inspiring to the users. The DISH concepts questionnaire also represents the psychological capability component of the meal decision questionnaire.
4. **Follow up of engagement and influence of DISH**
   1. **Environmental and Nutrition Scores (after):** intend to observe if the motivation persisted over the trial,
   2. **DISH Feedback:** have the participants' viewpoint of the DISH website and kiosk systems and gather feedback of the simulator for potential future enhancements.

**Table 1** shows the order in which these documents will be provided. All the information will be secured by encrypting the data, including personal scores and other responses. Authorized access will only be granted to members of the experimental design team.

***Table 1.*** *Order to provide the questionnaires and surveys.*

| **Study Timeline** | **Control** | **Treatment** |
| --- | --- | --- |
| Eligibility | 1 | 1 |
| Prior experiment | 2 | 2 |
| After phase one (website) | -- | 3 |
| After phase two (kiosk) | 4 | 4 |
